# Unbalanced Arginine pathway and altered maturation of pleural macrophages in Th2-deficient mice during *Litomosoides sigmodontis* filarial infection

**DOI:** 10.3389/fimmu.2022.866373

**Published:** 2022-10-24

**Authors:** Estelle Remion, Joséphine Gal, Soraya Chaouch, Jules Rodrigues, Nathaly Lhermitte-Vallarino, Joy Alonso, Linda Kohl, Marc P. Hübner, Frédéric Fercoq, Coralie Martin

**Affiliations:** ^1^ Unit Communication Molecules and Adaptation of Micro-organisms (MCAM, UMR 7245), Team Parasites and Free Protistes, Muséum National d’Histoire Naturelle, CNRS; CP52, 61 rue Buffon, 75005 Paris, France; ^2^ Institute for Medical Microbiology, Immunology & Parasitology (IMMIP), University Hospital of Bonn, Bonn, Germany; ^3^ German Center for Infection Research (DZIF), Partner Site Bonn-Cologne, Bonn, Germany

**Keywords:** parasite, nematode, filariasis, microfilaria, macrophage, lung, pleura

## Abstract

Filarial parasites are tissue dwelling worms transmitted by hematophagous vectors. Understanding the mechanisms regulating microfilariae (the parasite offspring) development is a prerequisite for controlling transmission in filarial infections. Th2 immune responses are key for building efficient anti-parasite responses but have been shown to also lead to detrimental tissue damage in the presence of microfilariae. *Litomosoides sigmodontis*, a rodent filaria residing in the pleural cavity was therefore used to characterize pleuropulmonary pathology and associated immune responses in wild-type and Th2 deficient mice. Wild-type and Th2-deficient mice (*Il-4rα^-/-^/Il-5^-/-^*) were infected with *L. sigmodontis* and parasite outcome was analyzed during the patent phase (when microfilariae are in the general circulation). Pleuropulmonary manifestations were investigated and pleural and bronchoalveolar cells were characterized by RNA analysis, imaging and/or flow cytometry focusing on macrophages. *Il-4rα^-/-^/Il-5^-/-^* mice were hypermicrofilaremic and showed an enhanced filarial survival but also displayed a drastic reduction of microfilaria-driven pleural cavity pathologies. In parallel, pleural macrophages from *Il-4rα^-/-^/Il-5^-/-^* mice lacked expression of prototypical alternative activation markers RELMα and *Chil3* and showed an altered balance of some markers of the arginine metabolic pathway. In addition, monocytes-derived F4/80^intermediate^ macrophages from infected *Il-4rα^-/-^/Il-5^-/-^* mice failed to mature into resident F4/80^high^ large macrophages. Altogether these data emphasize that the presence of both microfilariae and IL-4R/IL-5 signaling are critical in the development of the pathology and in the phenotype of macrophages. In *Il-4rα^-/-^/Il-5^-/-^* mice, the balance is in favor of parasite development while limiting the pathology associated with the host immune response.

## Introduction

The close relationship of filariae with their hosts has generated a complex balance between the host immune system, the induced pathology and the survival/transmission of the parasite. A modified immune response of the host may result in an enhanced or reduced survival of the parasite, altered ability to transmit the offspring, and can exacerbate or diminish the parasite-induced pathology. Any combination of the two components, parasites development and host pathology, is possible. Thus, in some cases although parasite survival is enhanced and parasite load is higher, parasite-induced pathology is reduced.

Several studies have analyzed how altering one or more cytokines can modulate the outcome of filarial infection. In particular, the murine filarial model *Litomosoides sigmodontis* has been extensively used to analyze the contribution of prototypic Th2 cytokines IL-4, IL-13 and IL-5 but also IFN-γ through the course of filarial infection (1–12). In this model, adult worms are located in the thoracic cavity between the visceral and the parietal pleura and females release blood circulating microfilariae during the patent phase of the infection (1, 2). By blocking signaling pathways associated with one or more of these cytokines, the development, survival and the transmission of the parasite is enhanced. Regarding IL-4, which signals through the same receptor as IL-13, studies have shown that the absence of this cytokine in susceptible BALB/c mice leads to an improved embryogenesis of the female filariae and an increased number of blood circulating microfilariae (3, 5, 6). The absence of IL-4 also turns resistant amicrofilaremic C57BL/6 mice into microfilaremic mice during *L. sigmodontis* infection (7). In BALB/c mice, the clearance of adult filariae is less efficient in mice depleted for IL-5 and the number of circulating microfilariae and frequency of microfilariae-positive animals is greater (4, 5, 8–10). Microfilaremia are equivalent in mice deficient for IL-4r or IL-5 and ten times higher than in wild-type (WT) mice (6). In *Il-4ra^-/-^/Il-5^-/-^* mice, microfilaremia is more than ten times higher compared to each single knock-out (KO) mice and all animals develop microfilaremia, in comparison to around 50% in WT mice (6, 12). Finally, the absence of IFN-γ also leads to a higher worm load but it does not impact the microfilaremia (4, 11). IFN-γ/IL-5 double-KO mice have a significantly higher worm load than any of the single-KO mice and a microfilaremia similar to that of IL-5 deficient mice (4).

In *Il-4ra^-/-^/Il-5^-/-^* mice, the balance is in favor of parasite development while at the same time limiting the pathology associated with the host immune response (12). *Il-4ra^-/-^/Il-5^-/-^* BALB/c mice are deficient for the α chain of the IL-4 receptor (IL4-Rα) and thus lacking IL-4/IL-13 signaling, leading to an absence of alternative activation of macrophages. Lack of IL-5 additionally impairs the maturation and recruitment of eosinophils (6, 12, 13). *Il-4ra^-/-^/Il-5^-/-^* mice present a strong susceptibility to *L. sigmodontis* filarial infection; they are hypermicrofilaremic and display reduced pleuropulmonary pathology in contrast to microfilaremic WT BALB/c mice. Indeed, WT mice show pleural, bronchoalveolar and lung-tissue inflammation associated with production of mucus, visceral pleura alterations (hyperplasia of cells) and fibrosis (12). The microfilaremic *ΔdblGata1* BALB/c mice, which lack the eosinophil lineage, exhibit similar pleural, bronchoalveolar and lung-tissue inflammation (12). Type 2 immunity characterized by increased production of the cytokines IL-4, IL-5, and IL-13 contributes to fibrosis following infection with helminth parasites including filariae (14). These cytokines also participate in the recruitment and activation of eosinophils and macrophages, which are the most numerous cells in the pleural cavity and in the broncho-alveolar space when *L. sigmodontis* infection occurs (2, 6, 12, 15).

Eosinophils are an essential component of the protective immune response. They contribute to the elimination of microfilariae, third-stage larvae and adult filariae through the formation of granulomas, extracellular traps (extracellular trap cell death, EETosis) or by the degranulation of their granules (cytotoxic molecules or enzymes) (10, 15–20). Interestingly, filariae accelerate their development in response to the IL-5-driven eosinophilia (21). Consequently, they release microfilariae earlier and in greater numbers (22).

The anti-helminth qualities of macrophages are also well-documented. However, the mechanisms they employ to promote the killing of filariae are not fully elucidated. Macrophages have been directly involved in microfilaria killing through nitric oxide (NO) production (23–26). They can also bind to larvae and adult worms, immobilize them and endorse the formation of large granuloma resulting in filarial killing (27, 28). Such granuloma formation is promoted by the Th2 cytokines IL-4, IL-5 and IL-13 which induce alternatively activated macrophages (AAMs) and eosinophil influx, thereby promoting activation of those AAMs and increased survival of eosinophils (29). AAMs also act to repair damaged tissue to ensure that parasite migration and resulting tissue damage is restricted and that wound healing is efficient (2, 30, 31). Previous studies have also shown that arginase (Arg) activity (32, 33), specifically Arg1 (34), is required for the anti-helminth properties of macrophages.

These macrophages have different origins. Tissues and organs are populated by resident macrophages (ResMac) that are maintained at homeostasis through self-renewal, independently of adult hematopoiesis (35–37). Macrophages can also be derived from monocytes, which are recruited to tissues during inflammatory processes. It is important to consider the ontogeny of these macrophages because their origin seems to influence the outcome of the *L. sigmodontis* infection (38). Indeed, a comparative analysis of the expansion dynamics of the mononuclear phagocyte system during pre-patent *L. sigmodontis* infection in susceptible BALB/c which allow a patent infection and resistant C57BL/6 mice that clear the infection shortly after the development of adult worms highlights striking differences between the strains. In C57BL/6 mice, the pool of resident macrophages is maintained independently of aging during *L. sigmodontis* infection. However, infected BALB/c mice fail to maintain the resident population by self-proliferation and need to be complemented by monocyte-derived macrophages (38).

Here, we investigated the two competing arginine pathways in macrophages, *i.e.* the metabolism *via* arginase or nitric oxide synthase and we further analyzed pleural and bronchoalveolar macrophage subsets to compare tissue-specific phenotypes during the patent phase of *L. sigmodontis* infection in low microfilaremic immunocompetent versus hypermicrofilaremic *Il-4ra^-/-^/Il-5^-/-^* BALB/c mice.

## Material and methods

### Mice and infestation protocol

Maintenance of the filaria *L. sigmodontis* Chandler, 1931 and isolation of infective larvae (L3) from the mite vector, *Ornithonyssus bacoti*, were carried out as previously described (39, 40). BALB/c OlaHsD mice were originally purchased from Envigo. The initial breeding pairs of *Il-4rα^-/-^/Il-5^-/-^* female BALB/c mice were kindly provided by Prof. Hübner (University Hospital Bonn, Germany). All mice were maintained and bred in the MNHN facilities on a 12-hour light/dark cycle. 6-8 weeks-old female mice were inoculated subcutaneously in the neck with a single dose of 40 L3.

### Dissection of mice, pleural and bronchoalveolar lavage, cell isolation and filarial load

Mice were sacrificed at 50 and 70 days post-inoculation (dpi). Filariae were collected with pleural cells by flushing the pleural cavity 10 times with 1ml cold phosphate buffered saline (PBS) as described in (41). Likewise, bronchoalveolar cells were collected by flushing 10 times the bronchoalveolar space with 1ml cold PBS as described in (41). The pleural and the bronchoalveolar lavage were both processed the same way: the first ml was isolated in a tube to limit fluid dilution and the remaining 9 ml were transferred to a second tube. The first ml of lavage in the first tube was centrifuged (5min, 250g, 4°C) and the cell pellet was added to the 9 ml of the second tube. The first ml was stored at -20°C for further analysis. The 9 ml were then centrifuged (5min, 250g, 4°C). Red blood cells were removed by hypotonic lysis and cells were diluted in 1 ml of PBS + 2% FCS and counted.

Filariae were counted, sexed and measured under a binocular microscope.

### Lung collection and sampling

After performing the bronchoalveolar lavage, lungs were exsanguinated. For this purpose, lungs and heart were removed from the thoracic cage and placed in a petri dish. A 23G needle was then inserted into the right ventricle of the heart to allow blood to flow out and 10ml of cold 1X PBS was injected into the left ventricle using another 23G needle. Post-exsanguination, lungs were inflated by injecting 1ml of ethanol 70% through the trachea. Lungs were preserved in ethanol 70% for microfilariae quantification by qPCR.

### Microfilaremia and purification of microfilariae

Peripheral, cardiac and pleural microfilariae were quantified at 70 dpi in a 10µl drop of blood or 10µl of the first ml of pleural fluid stained with Giemsa. Microfilariae present in the lung exsanguination fluid collected in a petri dish were transferred to a 15ml tube and centrifuged (15min, 500g). Red blood cells were removed by hypotonic lysis and microfilariae were diluted in 200µl of PBS and counted.

To isolate microfilariae from the general circulation, the protocol described in (12, 42) was used. Briefly, blood from mice was collected and the microfilariae were purified using a sucrose/Percoll density gradient, resuspended in 1mL PBS and counted using a hemocytometer (KOVA^®^ Glasstic Slide).

### Lung microfilariae detection and quantification

Lung DNA was extracted to quantify pulmonary microfilariae as described in (12). First, a 10 points standard curve was generated using lungs from naive mice to which a known number of microfilariae (from 0 to 1.000.000) was added before DNA extraction (see above for microfilariae purification).

Lungs from infected mice were homogenized in 500µl of PBS using a Tissue Lyser II (Qiagen). 100μl of homogenate solution was used for genomic DNA extraction (QIAamp DNA Mini Kit, Qiagen) according to the manufacturer’s protocol and finally eluted in 150μl of sterile water. A real-time PCR was performed with the SensiFAST TM SYBR^®^ No-ROX Kit (Bioline) in a LightCycler 480 (Roche Diagnostics) with an initial incubation of 10min (95 °C), 40 amplification cycles of 10 s (95 °C), 5 s (60 °C), and 10 s (72 °C), during which fluorescence data were collected. Filarial and murine DNA were detected by targeting *β-actin* of *L. sigmodontis* (*L.s.* actin 5’-GGCCGAACGTGAAATTGTACGTG-3’; 5’-GACCATCGGGCAATTCATACGACT-3’) and *β-actin* of *Mus musculus* (*M.m.* actin 5’-TGGAATCCTGTGGCATCCATGAAAC-3’; 5’-AGTCCGCCTAGAAGCACTTG-3’) respectively. For each sample, the ratio (R) of signal (CT) from filarial and murine *β-actin* was performed to normalize the results as R = CT (*L.s. actin*)/CT (*M.m. actin*).

The number of microfilariae in the lung of infected WT and *Il-4rα^-/-^/Il-5^-/-^* BALB/c mice was extrapolated using this ratio and the standard curve.

### Flow cytometry

Pleural and bronchoalveolar cells were washed twice in PBS prior to staining with LIVE/DEAD (Life Technologies) for 30 min at RT. Samples were blocked with murine Fc block CD16/CD32 before surface staining (20min on ice) with various specific fluorochrome-conjugated antibodies (see Supporting Information Table I for list of antibodies). For intracellular staining, samples were washed, permeabilized (FoxP3/Transcription Factor Staining Buffer Set, eBioScience, San Diego, CA) and stained for intracellular RELMα (Invitrogen, clone DS8RELM) for 30min.

Fluorescence Minus One (FMO) controls were used for each group with a pool of cells from all mice in the group. Cells were analyzed on a FACSVerse (BD Biosciences). Data was analyzed with FlowJo (FlowJo LLC). In order to compare macrophages population dynamics, samples were concatenated and a t-distributed stochastic neighbor embedding (tSNE) was performed on F4/80^+^ cells (gating strategy on **Figure 4A**).

### Isolation of mouse pleural macrophages

Pleural cells were distributed in 24-well plates (2.5x10^5^ for nitric oxide (NO) measurement and 5x10^5^ for arginase quantification), 6-well plates (10x10^5^) or 8-well Labtek chambered slides (Thermo Scientific) (1x10^5^), in RPMI, HEPES 25mM, 10% FCS, 1% penicillin/streptomycin and 2mM glutamine. Cells were allowed to adhere on the substrate for 2h (37°C, 5% CO_2_). Nonadherent cells were removed by gentle washing three times with warm PBS.

Depending on the assay, macrophages were either directly processed in each well (phagocytosis, arginase assay, quantification of collagen), lysed in Trizol (for subsequent RNA extraction) or detached (0.5ml Trypsin EDTA 1X during 5min at 37°C), counted and further cultured for 16h (NO measurement).

For arginase activity and quantification of collagen macrophages were cultured 24h in Iscove’s modified DMEM at 37°C, 5% CO_2_. Supernatants were collected and frozen at -20°C for quantification of collagen. 24h-cultured macrophages were lysed (5µl of 0.001% Triton X-100, 200µl of 25X proteases inhibitors and 4.8ml of distillated water, 15 minutes at room temperature under gentle shaking) and frozen at -20°C for arginase activity.

The macrophage purity was checked by flow cytometry using an anti-F4/80 antibody and was more than 90% (**Supplementary Figure 2A-C**).

### Quantification of collagen

100µl of bronchoalveolar lavage, pleural lavage or supernatants of 24h-cultured macrophages were added to a 96-well round bottom plate. 150µl of collagen coloring solution (0.1g Sirius red in 100 ml picric acid) was added to each well and incubated for one hour at 37°C. The plate was then centrifugated at 2 000g for 10 minutes and supernatants were removed. 100µl of absolute ethanol was added to each well and incubated for 2 minutes. The plate was centrifugated again for 10 minutes at 2 000g and supernatants removed. Pellets were resuspended in 200µl of 0.5M NaOH solution and incubated for 30 minutes at 37°C in the dark. The absorbance was read at 540nm and results were calculated with a two-fold dilution standard curve of collagen (Sigma-Aldrich, reference C9791-10MG) from 1mg/ml to 0.0078mg/ml.

### Measurement of nitrite production (Griess assay)

After inactivation of trypsin and washing with PBS, macrophages were resuspended in phenol-free RPMI, 10% FCS, 1% penicillin/streptomycin and 2mM glutamine and counted. Then macrophages were cultured for 16h in 96-well round bottom plates in triplicate (2.10^5^ macrophages/well/200µl) and stimulated with 20µg/ml IFN-γ or 10µg/ml filarial antigen. For *L. sigmodontis* antigen, female adult filariae were rinsed in PBS and homogenized in 500µl PBS using a Tissue Lyser II (Qiagen) for 1 minute at 30Hz, twice. The homogenate solution was sonicated in an ice bath at 40% of amplitude during 2 x 5 cycles of 10 seconds sonication with 10 seconds rest intervals. Insoluble material was removed by centrifugation at 300g for 10 min and 4°C (43). The protein concentration was determined with a spectrophotometer NanoDrop (Thermo Scientific). After 16h, the supernatants were harvested from each well for NO measurement. NO levels were determined by measuring its stable end product nitrite (NO_2_^-^). Briefly, equal amounts (100μl) of cell supernatants and Griess reagent (1% sulfanilamide, 0.3%N-1-naphthylethy-lenediamine dihydrochloride, 2.5% H_3_PO_4_) were blended and incubated (in the dark, 15min, RT). The optical density values of assay mixture were obtained by reading the absorbance at 540nm with a microplate reader (Labsystems Multiskan MS). Nitrite content was determined from a calibration curve plotted with a series of known concentrations of sodium nitrite (µM).

### Measurement of arginase activity

50µl of lysed macrophages were transferred in a new 96-well round bottom plate, 50µl of activation solution were added (10mM MnCl_2_, 50mM TrisCl, pH=7.5) and incubated for 10 minutes at 55°C. 25µl of each well were transferred in a new 96-well round bottom plate, 25µl of substrate solution (0.5M L-arginine, pH = 9.7) were added and incubated for one hour at 37°C. 50µl of each well were transferred in a new 96-well round bottom plate, 200µl of reagent from the QuantiChrom urea assay kit (BioAssay systems, reference DIUR-100) were added and incubated 20 minutes at room temperature in the dark. Absorbance was read at 520nm and results were calculated according to manufacturer’s instructions.

### Phagocytosis assay and multiplexed imaging of macrophages

A solution containing 5 µL of pHrodo™ Red E. coli BioParticles™ Conjugate (Invitrogen) in 100µL of complete medium was added to macrophages for 30 min (37°C, 5% CO2). Then 50µL of a solution containing fluorescently conjugated antibodies (1/400) and Hoechst (1/1000) was added; macrophages were further incubated for 30 min. Antibodies were the following: anti 1A/1E-BV421 (Biolegend, clone M5/114.15.2), anti CD11c-AF594 (Biolegend, clone N418), anti CD11b-PE (eBioscience, clone M1/70), and anti F4/80-APC (eBioscience, clone BM8). Macrophages were then washed twice with 100µL PBS to remove excess of particles and antibodies and fixed by adding 100µL of 4% PFA for 5min. Samples were washed twice with PBS then the wells from the Labtek slides were removed and samples were coverslipped using VectaMount (Vector Laboratories).

Slides were imaged on a Zeiss LSM880 confocal microscope as previously described (44). Acquisition was performed with a 32 channel Gallium arsenide phosphide (GaAsP) spectral detector using a 20× objective. Samples were excited simultaneously with 405, 488, 561 and 633 laser lines and signal were collected onto the linear array of 32 GaAsp detectors in lambda mode with a resolution of 8.9 nm over the visible spectrum. Spectral images were then unmixed with Zen software (Carl Zeiss) using reference spectra acquired from slides labelled with single fluorophores.

For cell segmentation, fluorescence signals from the membrane markers (CD11b, F4/80, MHCII and CD11c) were summed and the Cellpose algorithm (45) was applied using the online platform ZeroCostDL4Mic (46). Generated annotations were then imported in Imaris (Bitplane, version 9.9.1), surfaces were created for each annotation and different statistics were exported for cell shape (area and sphericity), cell surface markers (mean fluorescence intensity of CD11b, F4/80, MHCII and CD11c) and phagocytic capacity (mean fluorescence intensity of pHrodo bioparticles). A minimum of 600 cells were analyzed per mouse and the average value is displayed. In order to compare macrophage populations, fluorescence intensity values were imported into FlowJo, samples were concatenated and a t-distributed stochastic neighbor embedding (tSNE) was performed on CD11b^+^F4/80^+^ cells.

### Macrophage transcriptomic analysis

RNA extractions were performed with a phenol-chloroform solution. 200μl of chloroform was added in the Trizol solution containing cells and the sample was incubated (2min, RT) before being centrifuged (15min, 12 000g, 4°C). The aqueous phase containing RNA was transferred to a new tube and 500μl of 100% isopropanol was added. After 10min at RT, samples were centrifuged for (10min, 12 000g, 4°C). The RNA pellet was resuspended in 1ml of 75% ethanol and centrifuged (5min, 7500g, 4°C) prior to drying for at least 30min. Then RNA was eluted on ice with 40μl of RNase-free H_2_O. Finally, samples were incubated (10min, 55°C) and then kept on ice. RNA was quantified with a Qubit Fluorometer (Thermo Scientific). After treatment with DNase, complementary DNAs (cDNAs) were synthesized with Superscript^®^ IV reverse Transcriptase (Thermofisher). Transcripts of genes implicated in immune resolving and/or tissue repair were quantified by qRT-PCR with the LightCycler 480 II system and specific primers (see Supporting Information Table II for list of primer used). PCR amplification was analyzed by the E-ΔΔCT method and expression of the gene of interest was normalized by the expression of housekeeping genes *β-glucuronidase* and *β-actin*.

### Statistical analyzes

Representation and data analysis were performed with Prism 9.0 software (GraphPad Inc.). Data from independent experiments were pooled when possible. Data of microfilaremia (normally distributed) were analyzed with a Student’s t-test. All other results were analyzed by two-way ANOVA test to determine the effect of factors (group and/or time), followed by a Bonferroni’s multiple comparisons post-test when test application conditions were met (Gaussian distribution and homoscedasticity of the residuals). Otherwise a log or square transformation (depending on the skewness of the distribution of the variable) has been performed before the two-way ANOVA analysis. In all figures, the mean value is visually depicted. P values correlate with symbols as follows: *p<0.05, **p<0.01, ***p<0.001 represent differences between infected groups (naive, 50dpi, 70dpi); $p<0.05, $$p<0.01, $$$p<0.001 represent differences between mice strains (WT, *Il-4rα^-/-^/Il-5^-/-^*). Specific numbers of animals can be found in corresponding figure legends. Sketches were made using the Servier Medical Art image bank (https://smart.servier.com).

## Results

### Filarial development is improved in *Il-4rα^-/-^/Il-5^-/-^* mice

**Figure 1:**
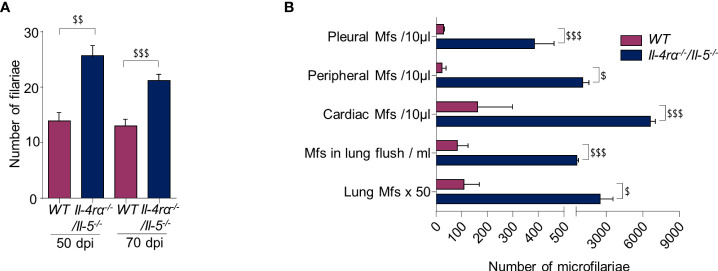
The absence of IL4R/IL5 signaling promotes filarial survival and production of microfilaria. WT and *Il-4rα^-/-^/Il-5^-/-^* BALB/c mice were infected with *L. sigmodontis* and parasitic load was determined at 50 and 70 days post infection (dpi). **(A)** Number of adult worms in the pleural cavity; results are expressed as mean ± SEM. n=9 WT and n=9 *Il-4rα^-/-^/Il-5^-/-^* mice at 50 dpi and n=24 WT and n=24 *Il-4rα^-/-^/Il-5^-/-^* mice at 70 dpi, pool of 1-3 experiments. **(B)** Number of microfilariae (Mfs) in pleural cavity fluid (n=8 per group of mice), in peripheral blood (n=24 per group of mice), in cardiac blood (n=24 per group of mice), in pulmonary flush (n=8 per group of mice) and in the whole lung tissue (n=5 per group of mice). For each location a t-test was performed ^$^p<0.05, ^$$^p<0.01 and ^$$$^p<0.001 represent differences between WT and *Il-4rα^-/-^/Il-5^-/-^* mice.

Filarial development was monitored in WT and *Il-4rα^-/-^/Il-5^-/-^* mice before and during the patent phase. Mice were inoculated subcutaneously in the neck with 40 *L. sigmodontis* L3. Consistent with previous studies (6, 12, 13), *Il-4rα^-/-^/Il-5^-/-^* mice had a higher worm burden in the pleural cavity in the patent phase of infection (70 dpi; **Figure 1A**). This difference was already visible at 50 dpi, a time point just before the filariae start to release microfilariae.

The microfilarial load of each mouse was evaluated at 70 dpi at different anatomical locations: the pleural cavity, the peripheral and cardiac blood, the pulmonary microcirculation and the lung tissue. Regardless of the location, and while all mice were microfilaremic, there was a higher number of microfilariae in *Il-4rα^-/-^/Il-5^-/-^* mice compared to WT mice (**Figure 1B**).

### Reduced pleuropulmonary pathology in *Il-4ra^-/-^/Il-5^-/-^* is associated with altered arginase pathway balance in pleural macrophages

**Figure 2:**
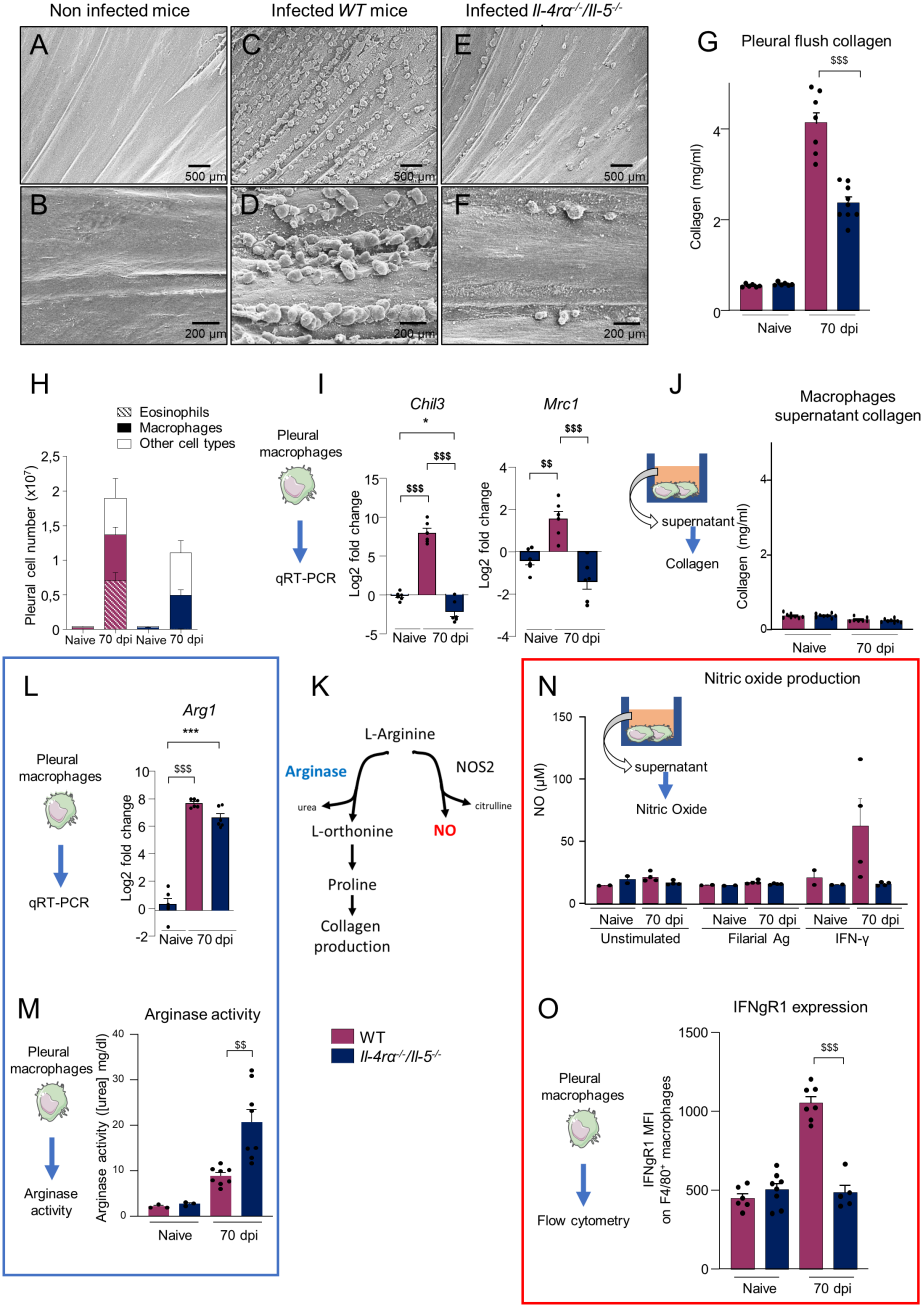
*Il-4rα^-/-^/Il-5^-/-^* mice showed decreased pleural pathology and altered balance of the arginine metabolic pathway. WT and *Il-4rα^-/-^/Il-5^-/-^* BALB/c mice were infected with *L. sigmodontis* and pleural cavity pathology and macrophages were investigated 70 days post infection (dpi). **(A–F)** Scanning Electron Microscopy images of the parietal pleura: **(A, B)** Smooth pleura in naive mice; **(F, I)** high density of polyps in 70 dpi WT mice, and **(E, F)** rare polyps in 70 days-infected *Il-4rα^-/-^/Il-5^-/-^* mice. **(G)** Collagen measurement in the pleural cavity flush. **(H)** Total number of F4/80^+^ macrophages and Siglec-F^+^ eosinophils as determined by flow cytometry. **(I)** Expression of prototypical markers of alternative activation *Chil3* and *Mrc1* in pleural macrophages determined by qRT-PCR. **(J)** Collagen measurement in macrophage culture supernatant after 24h culture *in vitro*. **(K)** Simplified arginine metabolic pathway, adapted from (47, 48). **(L)** Expression of *Arg1* by pleural macrophages determined by qRT-PCR. **(M)** *In vitro* arginase activity in pleural macrophages. Arginase activity was measured using QuantiChrom Arginase assay kit. **(N)** *In vitro* nitric oxide (NO) production (μM) from pleural macrophages after incubation for 16h with or without IFN-γ or filarial antigen (10µg/ml). **(O)** Mean fluorescence intensity (MFI) of IFNgR1 expression on F4/80^+^ pleural macrophages. Dots represent individual mice and results are expressed as mean ± SEM. **(A–F)** Images are representative of n=3 mice per group. **(H)** Pool of 4–6 independent experiments n=20-27 mice per group. **(J)** n = 10. **(I, L)**, n=6 per group. Two-way ANOVA: *p<0.05, ***p<0.001 represent differences between group; ^$$^p<0.01, ^$$$^p<0.001 represent differences between mice strains. **(N)** n=2-4 mice per group. **(O)** n = 8.

We recently described pathology of the visceral pleura (on the surface of the lung) in microfilaremic mice and gerbils (results from (12, 44) summarized in **Supplementary Table 1**). In the current study, electron microscopy of the costal parietal pleura showed a smooth appearance in naive mice (**Figures 2A, B**) while the parietal pleura of infected WT mice revealed a strong inflammatory reaction with a high density of polyps (**Figures 2C, D**) of similar aspect to those observed in gerbils (44). These polyps were mainly observed along the intercostal space, at the edge of the ribs. These pathological manifestations were reduced in 70 dpi *Il-4rα^-/-^/Il-5^-/-^* mice, which displayed only rare polyps (**Figures 2E, F**). Polyps and pleural hyperplasic areas were shown to be highly fibrotic (12, 44). In agreement with this, the amount of collagen in the pleural cavity lavage was higher in all infected mice and WT mice showed an even stronger response compared to *Il-4rα^-/-^/Il-5^-/-^* mice (**Figure 2G**).

A strong infiltration of cells occurs in the pleural cavity of mice during *L. sigmodontis* infection (12, 15). At 70 dpi, the pleural compartment of WT mice is mainly infiltrated by eosinophils and macrophages whereas in *Il-4rα^-/-^/Il-5^-/-^* mice, eosinophil infiltration is abrogated due to the absence of IL-5 and macrophages make up to 50% of pleural cells [**Figure 2H** and (12)]. The number of macrophages was similar in both groups of mice. As macrophages are important regulators of tissue healing and fibrosis (49), we explored their potential contribution to collagen production in the cavity. Pleural macrophages were enriched by adhesion to tissue culture plastic (**Supplementary Figures 2A-C**) and the expression of the prototypical alternative activation markers *Chil3* (Ym1) and *Mrc1* (CD206) was determined by qRT-PCR (**Figure 2I**). Both were strongly up-regulated in infected WT mice but not in *Il-4rα^-/-^/Il-5^-/-^* mice, confirming the absence of the IL4r*α*-driven alternative activation pathway of macrophages in *Il-4rα^-/-^/Il-5^-/-^* mice (6, 12, 13, 28).

No direct production of collagen by macrophages could be detected after 24h of *in vitro* culture of pleural macrophages (**Figure 2J**). However, macrophages can contribute to tissue repair or inflammation through differential metabolism of arginine [**Figure 2K** adapted from (47, 48)]. On one hand the action of Arginase1 (Arg1) leads to the formation of the proline necessary for collagen production (47, 48); on the other hand, nitric oxide synthase (NOS2) can use arginine to produce nitric oxide (NO) necessary for pathogen killing (47, 48). *Arg1* gene expression was highly upregulated in both pleural macrophages from WT and *Il-4rα^-/-^/Il-5^-/-^* mice (**Figure 2L**), as well as arginase activity *in vitro* (**Figure 2M**). Interestingly, while *Arg1* gene expression was similar between WT and *Il-4rα^-/-^/Il-5^-/-^* pleural macrophages, arginase activity was higher in the later (**Figure 2M**), suggesting a possible post-transcriptional regulation of Arg1.

NO production of WT and *Il-4rα^-/-^/Il-5^-/-^* pleural macrophages was then quantified *in vitro.* Cells were stimulated for 16h with filarial antigen or IFN-γ - which is increased in microfilaremic mice (12) and is a potent NO inducer (47, 48). Regardless of the group, unstimulated macrophages and macrophages stimulated with filarial antigen showed no increase of NO levels. Under IFN-γ stimulation, macrophages from WT 70 dpi infected mice produced NO while no NO production was induced in pleural macrophages of *Il-4rα^-/-^/Il-5^-/-^* mice (**Figure 2N**). Flow cytometry analysis of pleural cells for F4/80^+^ macrophages (**Supplementary Figure 2D**) showed that macrophages from *Il-4rα^-/-^/Il-5^-/-^* infected mice expressed similar levels of the IFN-γ receptor 1 (IFNgR1) to control mice whereas those from WT infected mice had high levels of IFNgR1 (**Figure 2O**).

Together, these results suggest that the arginine metabolism is affected in pleural macrophages during filarial infection and that this might contribute indirectly to collagen production and fibrosis in an Il-4rα/Il-5 dependent manner. In addition, *Il-4rα^-/-^/Il-5^-/-^* macrophages seems irresponsive to IFN-γ due to the absence of its main receptor.

### Pleural macrophages from *Il-4rα^-/-^/Il-5^-/-^* infected mice have increased phagocytic capacity but fail to mature into resident macrophages

**Figure 3:**
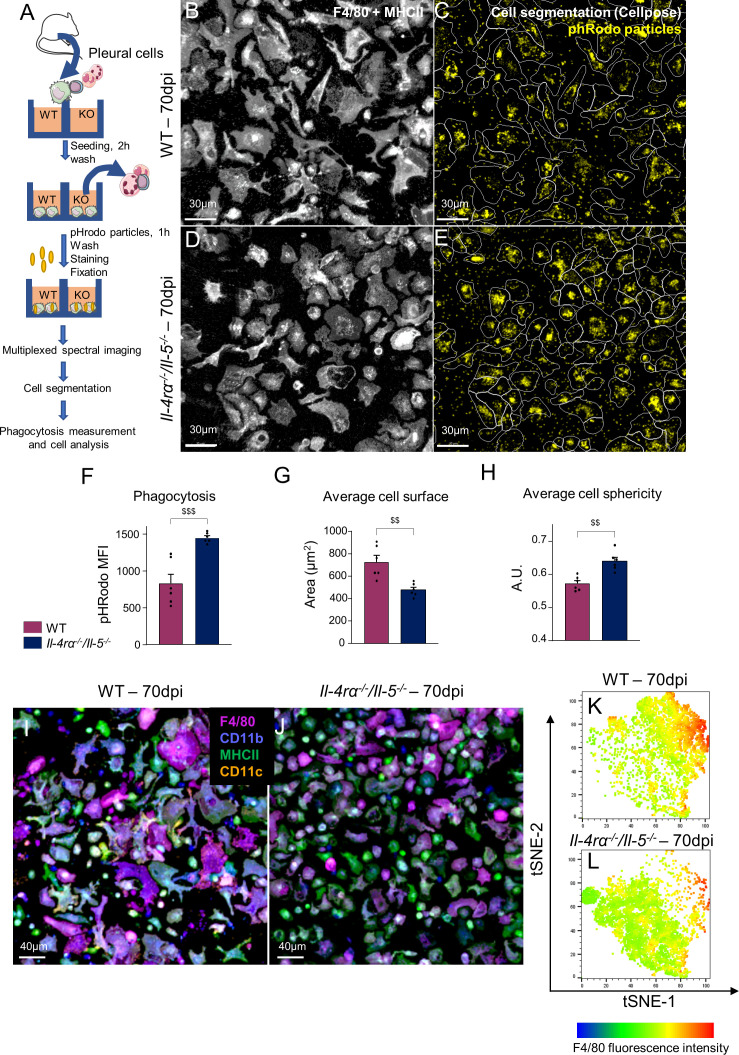
Pleural macrophages from Th2-deficient mice present higher phagocytic capacity but lower phenotypic diversity. Pleural macrophages from *L. sigmodontis* infected wild-type (WT) and *Il-4rα^-/-^/Il-5^-/-^* BALB/c mice were cultured with *E. coli* fluorescent bioparticles to address their phagocytic capabilities by confocal multiplex imaging. **(A)** Diagram of the experimental setup. **(B, D)** Aspect of the F4/80^+^ and/or MHCII^+^ adherent pleural cells from infected **(B)** WT and **(D)** *Il-4rα^-/-^/Il-5^-/-^* BALB/c mice. **(C, E)** Cellpose algorithm (45) cell segmentation outline (while line) and phRodo fluorescence signal (yellow) in infected **(B)** WT and **(D)** *Il-4rα^-/-^/Il-5^-/-^* BALB/c mice. **(F)** Phagocytosis expressed as phRodo mean fluorescence intensity (MFI). **(G)** Measure of the average cell surface. **(H)** Measure of the average cell roundness. **(I, J)** Multiplex images of adherent pleural macrophages from **(I)** WT and **(J)** *Il-4rα^-/-^/Il-5^-/-^* mice showing expression of F4/80 (purple), CD11b (blue), MHCII (green) and CD11c (orange). **(K, L)** tSNE analysis of single cell fluorescence intensity and shape parameters extracted from cell segmentation of microscopy images. Dots represent individual mice and results are expressed as mean ± SEM of n = 6 mice per group. For the tSNE analysis, at least 600 F4/80^+^ cells per mouse were concatenated. A t-test was performed (data normally distributed) ^$$^p<0.01 and ^$$$^p<0.001 represent differences between WT and *Il-4rα^-/-^/Il-5^-/-^* mice.

To further characterize the function of pleural macrophages in *Il-4rα^-/-^/Il-5^-/-^* infected mice, adherent macrophages were incubated with fluorescent pHrodo *E.coli* bioparticles to assess their phagocytic potential (**Figure 3A**). Pleural macrophages from *Il-4rα^-/-^/Il-5^-/-^* mice displayed a higher intensity of pHrodo fluorescence than their WT counterparts, indicative of higher phagocytic capacities (**Figures 3B-F**). Analysis of cell morphology in the same images indicated that macrophages from *Il-4rα^-/-^/Il-5^-/-^* mice were smaller (**Figure 3G**) and rounder (**Figure 3H**) than the WT ones, suggestive of a monocytic aspect. Multiplexed observation of the macrophages for F4/80, CD11b, MHCII and CD11c also suggested a higher phenotypic diversity in WT macrophages (**Figure 3I**) than in *Il-4rα^-/-^/Il-5^-/-^* (**Figure 3J**). Cell fluorescence intensity and shape information were extracted from the images to perform a t-distributed stochastic neighbor embedding (tSNE). Strikingly, this analysis highlighted a population of macrophages, mainly characterized by high levels of F4/80, which is present in WT infected mice (**Figure 3K**) and almost absent in *Il-4rα^-/-^/Il-5^-/-^* infected mice (**Figure 3L**).

**Figure 4:**
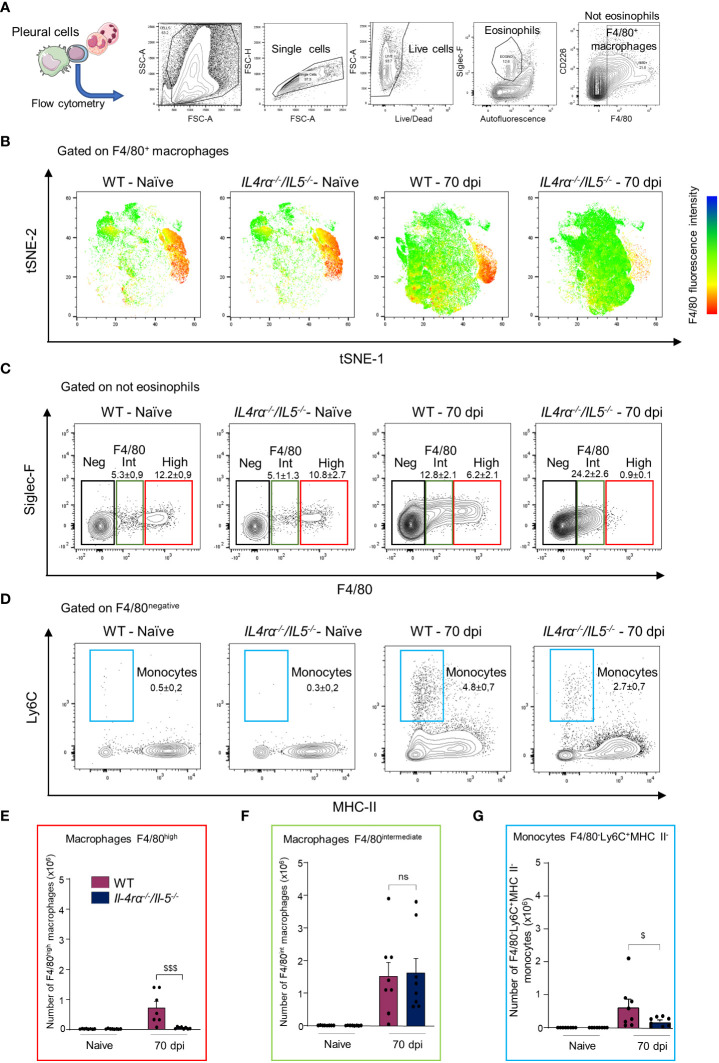
Pleural macrophages from *Il-4rα^-/-^/Il-5^-/-^* mice fail to mature into resident macrophages. Pleural cells were isolated from *L. sigmodontis* infected WT and *Il-4rα^-/-^/Il-5^-/-^* BALB/c mice at 70 dpi and analyzed by flow cytometry for F4/80, Ly6C, MHCII and Siglec-F. **(A)** Experimental setup and gating strategy. **(B)** Samples were concatenated and a tSNE was performed on F4/80^+^ cells. **(C)** Representative plots and gating strategy for F4/80^intermediate^ and F4/80^high^ macrophages and for naïve and infected WT and *Il-4rα^-/-^/Il-5^-/-^* BALB/c mice. An alternative gating strategy for F4/80^intermediate^ and F4/80^high^ including a CD11b and CD115 pre-gating is displayed in **Supplementary Figure 3**. **(D)** Representative plots and gating strategy for F4/80^-^MHCII^-^Ly6C^+^ monocytes. **(E)** Absolute numbers of F4/80^high^ macrophages. **(F)** Absolute numbers of F4/80^intermediate^ macrophages. **(G)** Absolute numbers of F4/80^-^MHCII^-^Ly6C^+^ monocytes. Dots represent individual mice and results are expressed as mean ± SEM of n = 8 mice per group. Two-way ANOVA: ^$^p<0.01, ^$$$^p<0.001 represent differences between mice strains, n = 8. ns, not significant.

We therefore analyzed further the pleural macrophage phenotype by flow cytometry (**Figure 4**). Again, a tSNE analysis of F4/80^+^ cells (comprising both F4/80^intermediate^ and F4/80^high^ macrophages) stained for Ly6C, MHCII and Siglec-F was performed on concatenated samples (**Figures 4A, B**) along with individual mice quantification (**Figures 4C-G**). Consistent with previous reports in BALB/c mice (36, 38, 50), pleural macrophages clustered into F4/80^high^ and F4/80^intermediate^ populations in naïve WT mice (**Figure 2B**). No difference could be observed in naïve WT or *Il-4rα* ^-/-^ */Il-5^-/-^* mice. Interestingly, in infected mice both F4/80^high^ and F4/80^intermediate^ were expanded in WT mice while *Il-4rα^-/-^/Il-5^-/-^* macrophages were most exclusively F4/80^intermediate^ (**Figures 4B, C, E, F** and **Supplementary Figure 3**). F4/80^intermediate^ macrophages have been shown to be monocyte-derived and serve to replenish the F4/80^high^ resident population in adult mice (36, 38). F4/80^-^Ly6C^+^MHCII^-^ monocytes were increased in both infected groups but they were higher in WT mice (**Figures 4D, G**).

Together, these data suggest that pleural macrophages from infected *Il-4rα^-/-^/Il-5^-/-^* mice display a more immature phenotype (smaller cells and F4/80 intermediate expression) and, in addition to the impairment of Th2 and arginase pathways (**Figure 2**), they fail to mature into F4/80^high^ resident macrophages (**Figures 3**, **4**).

### Monocyte-derived cells replenish the alveolar macrophage niche

**Figure 5:**
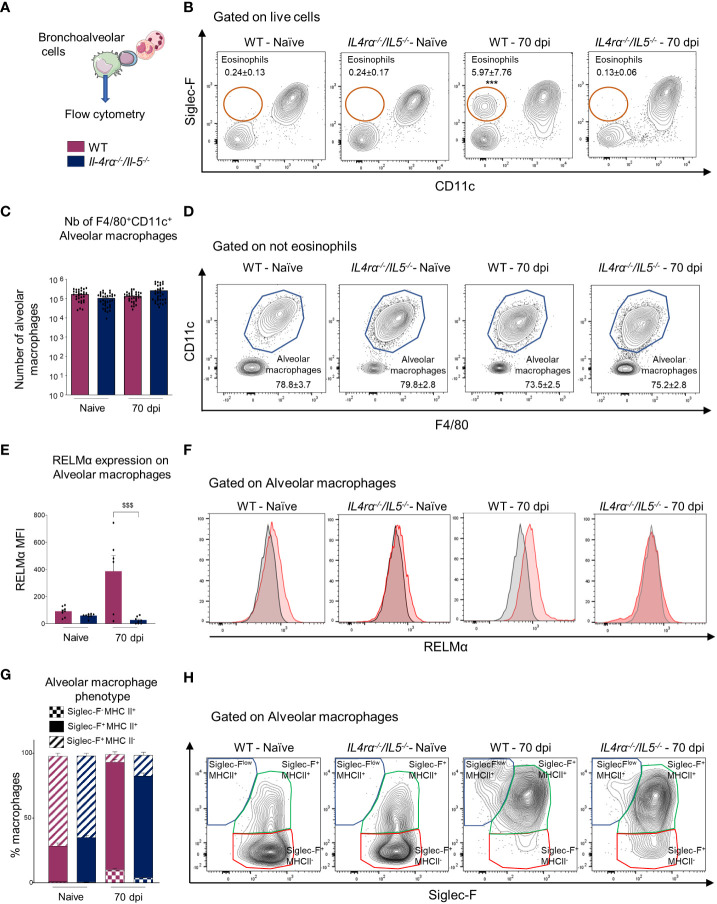
Monocyte-derived macrophages replenish the alveolar niche. Bronchoalveolar cells were isolated from naive or *L. sigmodontis* infected WT and *Il-4rα^-/-^/Il-5^-/-^* BALB/c mice at 70 dpi. CD11c^+^F4/80^+^ alveolar macrophages were analyzed by flow cytometry. **(A)** Experimental setup. **(B)** Representative plots and gating strategy for Siglec-F^+^ eosinophils. Average frequency +/- SEM of the different population is displayed. **(C)** Absolute number of CD11c^+^ F4/80^+^ alveolar macrophages. **(D)** Representative plots and gating strategy for CD11c^+^F4/80^+^ alveolar macrophages. Average frequency +/- SEM of the different population is displayed. **(E)** Mean fluorescence intensity (MFI) of RELMα expression on CD11c^+^ F4/80^+^ alveolar macrophages. RELMα expression was normalized by subtracting the Fluorescence Minus One (FMO) control MFI. **(F)** Representative expression of RELMα by CD11c^+^ F4/80^+^ alveolar macrophages. Shaded histograms represent FMO controls. **(G)** Average frequency of Siglec-F^low^ MHC^+^, Siglec-F^+^MHC^+^ and Siglec-F^+^MHC^-^ cells among CD11c^+^ F4/80^+^ alveolar macrophages. **(H)** Representative plots and gating strategy for Siglec-F^low^ MHC^+^, Siglec-F^+^ MHC^+^ and Siglec-F^+^ MHC^-^ cells among CD11c^+^ F4/80^+^ alveolar macrophages. Dots represent individual mice and results are expressed as mean ± SEM. **(C)** n=30-34 naive and n=20 infected mice per group, pool of 6 independent experiments. **(E)** n=6-8 mice per group, pool of 2 independent experiments. **(G)** n=10-16 mice per group, pool of 3 independent experiments. Two-way ANOVA: ^$$$^p<0.001 represent differences between mice strains.

We finally aimed to explore whether alveolar macrophages were affected in the same way during the infection. Indeed, microfilariae are found in high number in the lung circulation and affect both bronchoalveolar and perivascular spaces (12). Bronchoalveolar cells were analyzed by flow cytometry for F4/80, Siglec-F, CD11c, RELM*α* and MHCII (**Figure 5A**). As already reported (12), eosinophils were highly increased in the bronchoalveolar space of WT infected mice and this was abrogated in *Il-4rα^-/-^/Il-5^-/-^* animals (**Figure 5B**). The number of alveolar macrophages was similar between naïve and infected mice, irrespective of the genetic background (**Figures 5C, D**). However, RELMα, a marker of alternative activation of macrophages (28) was increased in alveolar macrophages from WT infected mice but not in those from *Il-4rα^-/-^/Il-5^-/-^* mice, confirming the absence of Th2 responses in these cells (**Figures 5E, F**). It was recently demonstrated by McCowan et al. (2021) (51) and others (32, 52) that upon inflammation, alveolar macrophage renewal occurs through recruitment of monocytes which then mature into alveolar macrophages (CD11c^+^ Siglec-F^+^ cells). These newly arrived monocyte-derived alveolar macrophages were shown to transiently express MHCII and low-intermediate levels of Siglec-F (51). In the *L. sigmodontis* filarial model, the percentage of Siglec-F^low^ MHC^+^ and Siglec-F^+^ MHC^+^ alveolar macrophages were significantly increased in both groups of infected animals (**Figures 5G, H**), suggesting an active replenishment of the alveolar niche by bone marrow derived monocytes. However, when comparing infected WT and *Il-4rα^-/-^/Il-5^-/-^* mice, the percentage of Siglec-F^low^ MHC^+^ was lower and Siglec-F^+^ MHC^-^ was higher in Th2-deficient mice, suggesting a faster acquisition of a bona fide macrophage phenotype in *Il-4rα^-/-^/Il-5^-/-^* mice.

Together, these results suggest that even in the absence of Th2 immune response, *Il-4rα^-/-^/Il-5^-/-^* mice maintain the ability to replenish the alveolar niche with monocyte-derived cells upon infection and suggest that alveolar macrophages from *Il-4rα^-/-^/Il-5^-/-^* mice could mature faster than their WT counterpart.

## Discussion

Th2 immune responses are essential to parasite killing, but also tissue repair and fibrosis (14, 53). Using the model of filarial infection *L. sigmodontis*, we and others have shown that major alteration of Th2 responses in *Il-4rα^-/-^/Il-5^-/-^* BALB/c mice favors dramatically parasite development, survival and reproduction (6, 13, 28). Due to the absence of eosinophils and alternative activation of macrophages, these mice present extremely high microfilaremia (the presence of parasite offspring in the circulation), higher adult parasite burden and increased worm size (6, 12, 13, 28). Adult filariae reside in the pleural cavity, where they mate and release microfilariae about 60 days after the infection (54). Pleural effusion and increased pleural immune cell numbers are therefore a common feature of the infection in C57BL/6 and BALB/c mice (2, 12, 41, 50). In addition, in immunocompetent rodent hosts the presence of *L. sigmodontis* adult worms and microfilariae in the pleural cavity induces a large variety of pleuro-pulmonary pathologic manifestations. In microfilaremic BALB/c mice, we previously described fibrotic hyperplasic lesions on the visceral pleura while in gerbils, polypoid structures were found at the surface of the lung (12, 44). In the present study, we describe similar polypoid structures on the parietal pleura of BALB/c mice, along the edge of the ribs. Their absence from the visceral pleura in mice suggests unique anatomical features of the intercoastal groove. In addition to being highly vascularized, the intercostal groove (unlike the visceral pleura) displays stomata, which are connected to collector lymphatic vessels. Pleural fluid and inflammatory pleural cells are permanently drained through these stomata (55, 56). The exit way of microfilariae from the pleural cavity is unknown but stomata could provide the perfect backdoor to reach general circulation. Another option would be for microfilariae to directly cross the mesothelial layer to reach sub-pleural blood vessels (57). In both cases, microfilariae driven-local inflammation could explain the pattern of localization of the polyps. The composition of polyps has not yet been characterized in mice, but it has been shown in gerbils that they are mainly composed of immune cells, especially lymphocytes, eosinophils and macrophages (44). These polyps on the lung surface are fibrotic and could be the result of a slight uplift of the mesothelium followed by a proliferative reaction of the visceral pleura (44). Interestingly, the formation of microfilariae-driven pleural lesions is greatly reduced in the absence of eosinophils (in *Il-4rα^-/-^/Il-5^-/-^* and *ΔdblGata)* (12). When stimulated by filarial parasites, eosinophils unleash their cytotoxic granules (MBP, EPO, etc.) (20, 58, 59) and Mfs have been shown to induce the formation of Eosinophil Extracellular Traps (EETs) (16) similar to their neutrophil counterpart, the NETs (neutrophil extracellular traps) (60). Eosinophils, through antibody-dependent cell-mediated cytotoxicity (ADCC) (16, 18, 20, 61–65) or EETs (16) are important to kill microfilariae but they could also induce collateral damage to the pleura and therefore initiate the pathologic process. However, it is unlikely that eosinophils are the only mediators of pleural pathology.

Along with eosinophils, macrophages are the major infiltrates in pleural effusions in WT mice and are the main cells present in *Il-4rα^-/-^/Il-5^-/-^* mice. We therefore decided to explore how macrophages are affected in the altered Th2 context of *Il-4rα^-/-^/Il-5^-/-^* mice and whether they could also participate to the initiation and maintenance of tissue fibrosis (47, 48, 66). Pleural pathology was mirrored by high quantities of free collagen in the cavity flush, probably a signature of the fibrotic environment. Pleural macrophages were not producing collagen *in vitro* but it is known that through differential metabolism of arginine, macrophages can orchestrate inflammation and tissue repair (66).

Arginine can be metabolized by Arginase 1 (Arg1). Arginase 1 expression is induced by anti-inflammatory signals such as IL-4, IL-13, IL-10 or TGFβ (47, 48, 66, 67), which are present in high quantity in the pleural cavity of *L. sigmodontis* infected mice (6, 12, 15, 68, 69). Arginase activity leads to the production and release of proline, necessary for collagen synthesis. It has therefore been associated with tissue repair but also, in cases of unresolved inflammation, with tissue fibrosis (47, 48, 66). WT macrophages expressed high level of *Arg1* transcripts and activity. Arg1 has been widely used as a maker of Th2-driven alternatively activated of macrophage. In our hands, macrophages from *Il-4rα^-/-^/Il-5^-/-^* lack the prototypical makers of alternatively activated macrophages Chil3 and Mrc1 but display high levels of Arg1. This highlights previous reports indicating that Arg1 can be induced independently of IL4R signaling and supports the use of multiple markers when assessing macrophage activation status (70, 71).

Through the action of the nitric oxide synthase (NOS), arginine can also be metabolized to nitric oxide (NO) and citrulline (47, 48, 66). NOS activity is increased by inflammatory signals such as IFNγ, TNFα or IL1β (66). Microfilariae induce a strong IFNγ response (6, 11, 12) and this has been associated with a mixed Th1/Th2 phenotype of pleural cells (12, 72–74). WT pleural macrophages from infected mice are able to produce NO *in vitro* when stimulated with IFN-γ, suggesting that NO must be produced *in vivo* in the pleural cavity. Previous reports indicated that even if NO can kill Mfs *in vitro*, inhibition of nitric oxide synthesis or use of NOS-deficient mice did not affect microfilaremia *in vivo* (75, 76). However, if NO is inefficient at killing parasites, it could lead to tissue detrimental oxidative damage. It was also hypothesized that NO can modulate permeability of the visceral and parietal pleurae as well as leading to a wider opening of the stomata (77, 78). NO could therefore help microfilariae to escape from the pleural cavity. Surprisingly, macrophages from *Il-4rα^-/-^/Il-5^-/-^* mice were unable to produce NO when stimulated with IFN-γ, and this was associated with the low cell expression of the IFN receptor (IFNgR1), suggesting that a competent Th2 signaling in pleural macrophages is important for inducing inflammatory responses. However, when cultured with bacterial bio-particles, Th2-deficent macrophages displayed increased phagocytosis capacities compared to their WT counterparts. Live microfilariae and adult worms are too big and fast-moving to be directly phagocytosed (79). It is therefore likely the increased phagocytosis in *Il-4rα^-/-^/Il-5^-/-^* mice does not confer a competitive advantage.

Together, the production of NO through NOS and proline through Arg1 could respectively induce tissue damage and provide the metabolites necessary for the production of collagen. A lack of NO-driven tissue damage in *Il-4rα^-/-^/Il-5^-/-^* mice could therefore be a reason for decreased pathology in these animals. Arg1 and NOS2 compete for L-arginine arginine, so a disequilibrium towards one side could result in the higher Arg1 activity in *Il-4rα^-/-^/Il-5^-/-^* mice. Interestingly, a recent report in a pulmonary nematode infection (*Nippostrongylus brasiliensis*) showed that alveolar macrophages can mediate parasite killing by locally deleting L-arginine through Arg1 (32). However, this competition for metabolites does not seem efficient in the pleural cavity as while Th2 deficient mice show a higher Arg1 activity, they still allow a higher parasite development. Future work will decipher whether macrophages from infected animals are able to undergo both Arg and NOS pathways in the same cells or if different macrophages populations are following only one way. Indeed, multiplex imaging and flow cytometry of pleural macrophages indicated a higher phenotypic diversity in WT mice than in *Il-4rα^-/-^/Il-5^-/-^* mice. In naïve BALB/c mice, pleural macrophages are divided into large F4/80^high^ resident macrophages (ResMac) and small F4/80^intermediate^ monocyte-derived macrophages (MoMac), the latter being able to replenish the F4/80^high^ pool ResMac (36, 38, 50). During *L. sigmodontis* infection, it was previously shown that macrophage increase is due to influx of Mo-Macs in BALB/c mice (38, 50). Interestingly, if the phenotype and distribution of pleural macrophages in naïve WT and *Il-4rα^-/-^/Il-5^-/-^* mice (with ResMac and MoMac) was similar, the ResMac population was almost absent in *Il-4rα^-/-^/Il-5^-/-^* mice. This suggests that, in absence of potent Th2 immune responses, MoMacs fail to mature into ResMac. The absence of difference in naïve mice could be due to the fetal origin of the initial pool of pleural macrophages (36), which would not need Th2 signaling. Similar results were recently observed by Finlay et al. (50) before patent *L. sigmodontis* infection. This suggests that microfilariae release is not able to restore monocyte to macrophage transition in the absence of IL4r*α*. Interestingly, Finlay et al. and previous work from their lab (38, 50) also showed major differences in macrophage dynamics between infection resistant C57BL/6 mice and BALB/c mice. Indeed, MoMac to ResMac conversion was very efficient in C57BL/6 mice compared to BALB/c mice. In addition, ResMac from C57BL/6 mice were able proliferate locally during infection while BALB/c macrophages couldn’t. Impairing IL4rα signaling in C57BL/6 mice reduced their ability to convert MoMac into ResMac and this was associated with increased survival of parasites (50). Together our results and the previously mentioned ones suggest that F4/80^high^ ResMac are important for parasite control whereas MoMac are rather inefficient. Interestingly, in the bronchoalveolar space, IL4Rα/IL5 deficiency does not seem to prevent recruitment of monocytes and their maturation to alveolar macrophages. McCowan and al recently showed that the transcription factor EGR2 is necessary for repopulation of the alveolar macrophage niche by monocytes, and that its expression was independent of IL4R signaling (51). The fact that we still observed slight differences in the proportion of mature and transitioning alveolar macrophages in WT and Th2-deficient mice could be due to eosinophil-derived factors.

If F4/80^high^ pleural macrophages are necessary to clear *Litomosoides sigmodontis* infection, cavity macrophages can also have important roles in mesothelial damage healing and pathology, as highlighted by elegant work by Kubes’ laboratory using intravital microscopy of peritoneal cavity macrophages (80, 81). It was shown that peritoneal F4/80^high^ GATA6^+^ macrophages can tether and aggregate to injuries in a scavenger receptor-dependent manner and promote repair of lesions (81). Interestingly, F4/80^low^ MoMac were shown to be unable to adhere to lesions (82). In a model of surgery-induced adhesions, F4/80^high^ macrophages formed aggregates leading to the formation of fibrotic scar tissue in the peritoneal cavity (81). A similar process could be at the origin of the formation of the polyps observed in the pleural cavity of microfilaremic mice and gerbils and the low number of F4/80^high^ macrophages in *Il-4rα^-/-^/Il-5^-/-^* mice could impair the formation of pleural pathology. This is supported by the fact that granulomas (aggregates of myeloid cells around parasites (27)) were never observed at 70 dpi in *Il-4rα^-/-^/Il-5^-/-^* mice, suggesting that these macrophages have altered adhesion properties.

**Figure 6:**
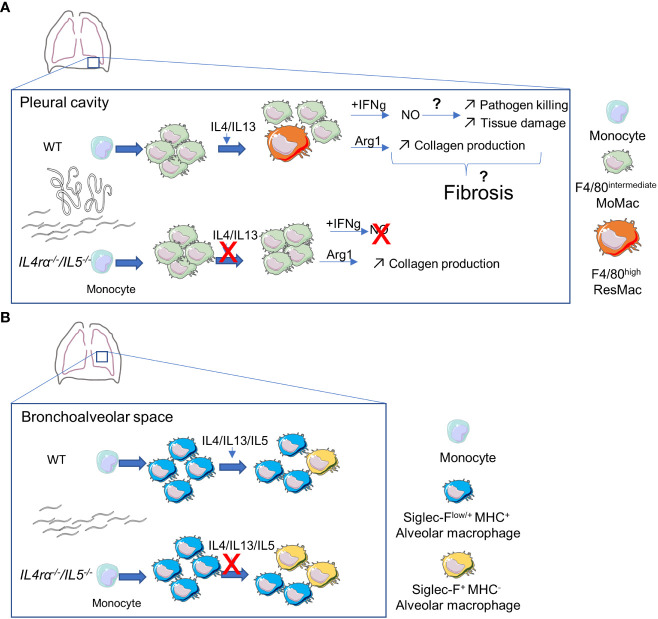
Potential effects of altered Th2-immune responses on pleural and alveolar macrophages dynamics and functions during patent *Litomosoides sigmodontis* infection. **(A)** In the pleural cavity, adult worms and microfilariae induce a strong recruitment of F4/80^intermediate^ Monocyte-derived macrophages (MoMac). Some of them will then mature into F4/80^high^ resident macrophage (ResMac) in an IL4R-dependent manner. Macrophages acquire an IL4R-driven alternative activation state and increase Arginase1 (Arg1) expression and activity. This might lead to increased proline availability and collagen production in the cavity. On the other hand, pleural macrophages upregulate IFNgR1 and in the presence of IFNγ, macrophages produce nitric oxide (NO). NO could then favor pathogen killing but also tissue damage. Together, tissue damage and increased collagen could favor tissue fibrosis. In the absence of IL4-receptor and IL5, MoMac are still recruited but fail to mature into ResMac. IFNgR1 expression is not upregulated leading to an unresponsiveness to IFNγ impairing NO production. Arg1 expression is not affected and its activity is increased compared to WT mice, but, maybe because of the absence of tissue damage, this is not sufficient to initiate tissue fibrosis. **(B)** In the bronchoalveolar space, monocyte-derived Siglec-F^low/+^ MHC^+^ alveolar macrophages seem to repopulate the Siglec-F^+^ MHC^-^ *bona fide* alveolar macrophage population. In the absence of Th2-immune responses and eosinophils, the maturation of these transient macrophages could be faster.

As a conclusion, the infection of *Il-4rα^-/-^/Il-5^-/-^* mice highlights the subtle balance necessary to control infections while maintaining tissue homeostasis (**Figure 6**). Indeed, on one hand *Il-4rα^-/-^/Il-5^-/-^* mice show very little tissue damage and pathologic lesions. This is probably due to the combination of many factors including the absence of eosinophilic inflammation as well as impaired alternative activation and maturation of macrophages. On the other hand, the same cells (eosinophils and F4/80^high^ ResMac) are the most efficient cells to kill adult parasites and microfilariae. *Il-4rα^-/-^/Il-5^-/-^* mice, as they lack both eosinophils and ResMac therefore allow an exceptional survival, growth and reproduction of the parasite. We need a holistic view of filarial infection to better understand the pathogenesis mechanisms and target specific pathways to maintain tissue integrity while allowing efficient parasite killing.

## Data availability statement

The raw data supporting the conclusions of this article will be made available by the authors, without undue reservation.

## Ethics statement

All experimental procedures were carried out in accordance with the EU Directive 2010/63/EU and the relevant nationallegislation, namely the French “Décret No. 2013-118, 1er février 2013, Ministère de l’Agriculture, de l’Agroalimentaire et de la Foret”. Protocols were approved by the ethical committee of the Museum National d’Histoire Naturelle (CEEA 68 Cuvier, Project agreement #13845) and by the Direction Départementale de la Cohésion Sociale et de la Protection des Populations (DDCSPP) (No. D75-05-15).

## Author contributions

CM, FF and ER contributed to conception and design of the study. Investigation: ER, JG, JR, SC, NL-V, JA, FF and CM. Formal analysis: ER, JG, and CM. Statistics: ER, JG and CM. Writing – original draft: ER, JG and CM. Writing – review & editing: ER, JG, LK, MH, FF and CM. All authors contributed to manuscript revision, read, and approved the submitted version. All authors contributed to the article and approved the submitted version.

## Funding

Core funding from the Museum National d’Histoire Naturelle. European Community grant H2020-EU.3.1.3.-HELP-815628. French Agence Nationale de la Recherche (ANR) grant, Project WOLF (ANR-21-CE13-0029).

## Acknowledgments

We thank Geraldine Toutirais from the MNHN Electron Microscopy facility (PTME, Plateau Technique de Microscopie Electronique et de Microanalyses du Museum National d’Histoire Naturelle, Paris) for assistance with SEM imaging. We thank Cyril Willing from the MNHN light microscopy facility (CeMIM, Centre de Microscopie et d’IMagerie numerique, MNHN Paris) for assistance with confocal imaging.

## Conflict of interest

The authors declare that the research was conducted in the absence of any commercial or financial relationships that could be construed as a potential conflict of interest.

## Publisher’s note

All claims expressed in this article are solely those of the authors and do not necessarily represent those of their affiliated organizations, or those of the publisher, the editors and the reviewers. Any product that may be evaluated in this article, or claim that may be made by its manufacturer, is not guaranteed or endorsed by the publisher.

## Supplementary material

Supplementary Figure 1 Overview of pleuropulmonary pathologies in WT and *Il-4rα^-/-^/Il-5^-/-^* mice. The first column indicates the localization of the observed pathology. The second indicates the pathology. The following columns compare naive WT mice to infected mice at 70 dpi and gerbils at 70 dpi (–). means that the pathology is absent, (+) indicates a low pathology, (++) a medium pathology and (+++) a high pathology, ND = not determined.

Supplementary Figure 2 Supplementary information for . **(A)** Experimental setup for the viability test of macrophages. **(B)** Gating strategy for live cells and F4/80^+^ macrophages. **(C)** Average frequencies of live cells and F4/80^+^ macrophages after 24h culture. **(D)** Gating strategy for F4/80^+^ macrophages used for the IFNgR1 MFI (related to ).

Supplementary Figure 3 Alternative gating strategy for F4/80^high^ and F4/80^low^ macrophages. **(A)** Gating strategy including a pre-gating of CD115 and CD11b (related to ). **(B)** Number of F4/80^intermediate^ macrophages (related to ). **(C)** Number of F4/80^high^ macrophages (related to ).
