# Supplementary figures and images for: Unbalanced Arginine pathway and altered maturation of pleural macrophages in Th2-deficient mice during Litomosoides sigmodontis filarial infection
Source: Front Immunol. 2022 Oct 24;13:866373. doi: 10.3389/fimmu.2022.866373 (PMC9637854; doi:10.3389/fimmu.2022.866373)

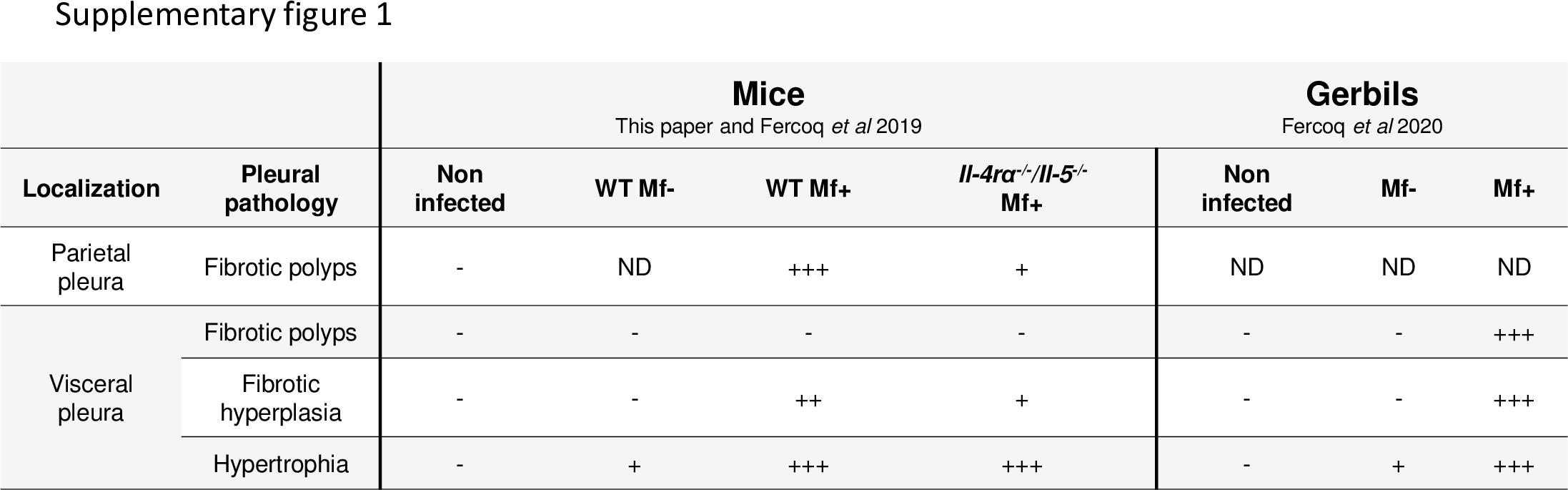

Supplement: Supplementary Figure 1 — Overview of pleuropulmonary pathologies in WT and Il-4rα-/-/Il-5-/- mice. The first column indicates the localization of the observed pathology. The second indicates the pathology. The following columns compare naive WT mice to infected mice at 70 dpi and gerbils at 70 dpi (–). means that the pathology is absent, (+) indicates a low pathology, (++) a medium pathology and (+++) a high pathology, ND = not determined. [file Image_1.tif]

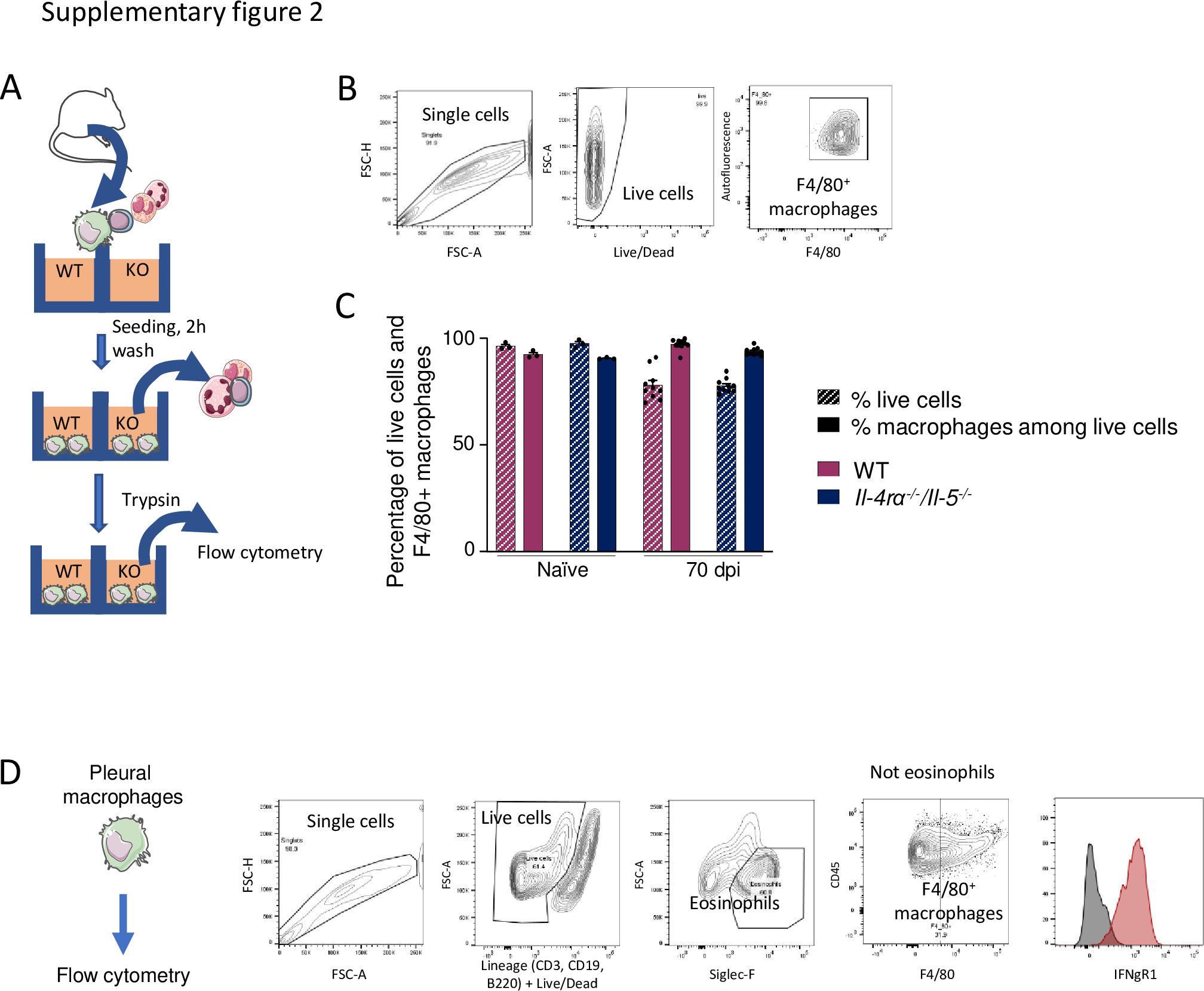

Supplement: Supplementary Figure 2 — Supplementary information for . (A) Experimental setup for the viability test of macrophages. (B) Gating strategy for live cells and F4/80+ macrophages. (C) Average frequencies of live cells and F4/80+ macrophages after 24h culture. (D) Gating strategy for F4/80+ macrophages used for the IFNgR1 MFI (related to ). [file Image_2.tif]

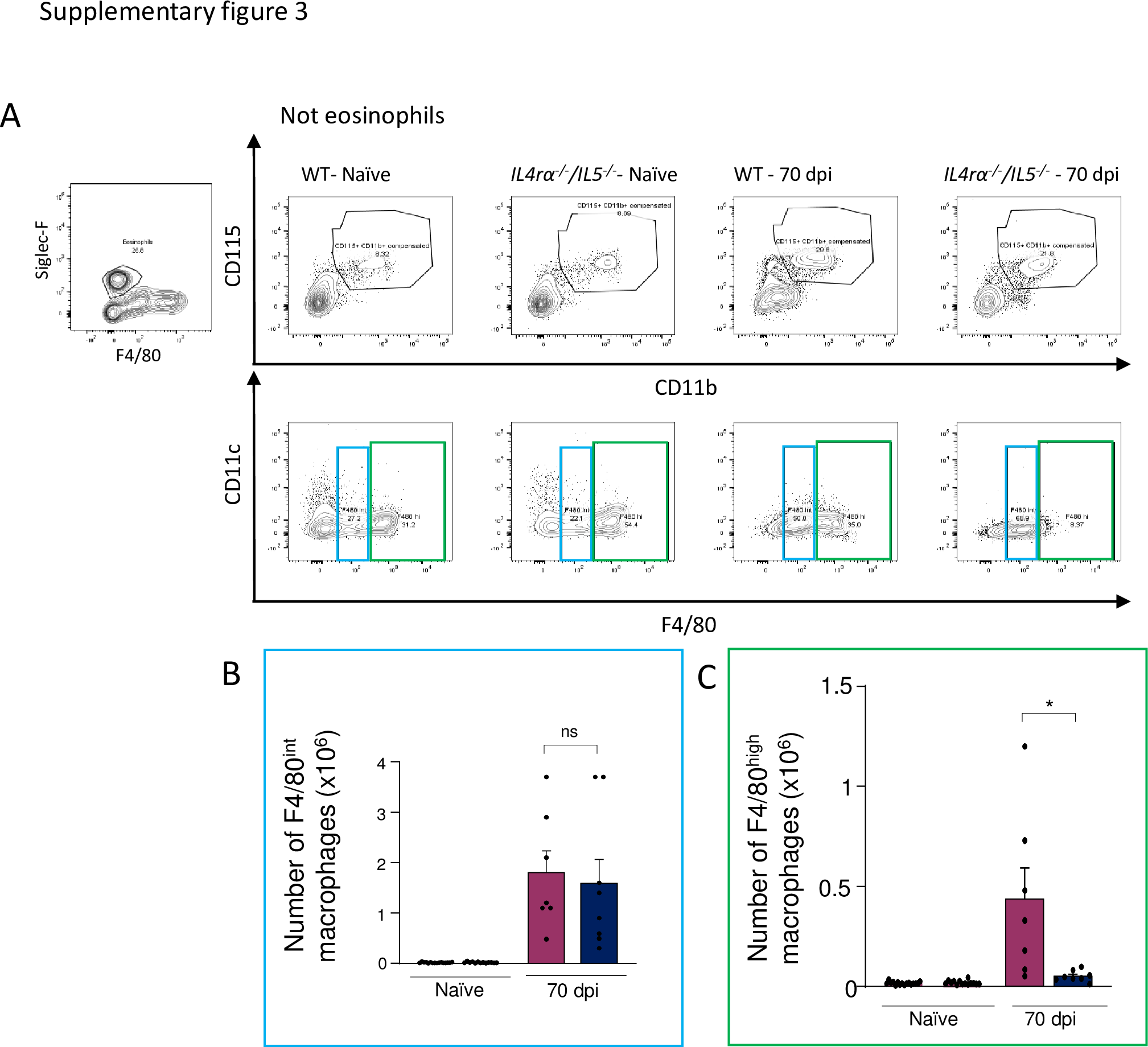

Supplement: Supplementary Figure 3 — Alternative gating strategy for F4/80high and F4/80low macrophages. (A) Gating strategy including a pre-gating of CD115 and CD11b (related to ). (B) Number of F4/80intermediate macrophages (related to ). (C) Number of F4/80high macrophages (related to ). [file Image_3.tif]
